# Correlation between F18-FDG PET/CT Imaging and BRAF V600E Genetic Mutation for the Early Assessment of Treatment Response in Papillary Thyroid Cancers

**DOI:** 10.3390/jpm10020052

**Published:** 2020-06-19

**Authors:** Andra Piciu, Maria-Iulia Larg, Doina Piciu

**Affiliations:** 1 Department of Medical Oncology Iuliu Hațieganu, University of Medicine and Pharmacy, 400012 Cluj-Napoca, Romania; piciuandra@gmail.com; 2 Department of Endocrine Tumors and Nuclear Medicine, Institute of Oncology “Prof.dr.Ion Chiricuță” 400015 Cluj-Napoca, Romania; doina.piciu@gmail.com; 3 PhD School Iuliu Hațieganu University of Medicine and Pharmacy, 400012 Cluj-Napoca, Romania

**Keywords:** papillary thyroid cancer, SUV PET/CT, BRAF V600E

## Abstract

In thyroid neoplastic pathology, the BRAF V600E mutation is shown to be involved in the oncogenesis of papillary thyroid cancer and its subtypes. The purpose of this study is to evaluate the correlation between the mutation of the BRAF V600E oncogene and the pathological standardized uptake values (SUV) at the F18-fluorodeoxyglucose (F18-FDG) positron emission tomography/computed tomography (PET/CT) evaluation, for a group of 20 patients with radically treated (total thyroidectomy and radioiodine therapy) papillary thyroid cancer, with subclinical persistent disease, at 6 months after the initial treatment. We analyzed the correlations between the values of SUV and the presence of the BRAF mutation as well with other prognostic factors such as stage, age, specific tumor markers (thyroglobulin and anti-thyroglobulin), extrathyroid extension, the presence of metastatic lymph nodes or distant metastasis. The value of SUV in the case of BRAF+ (positive) patients was higher than in the negative ones, but without statistical significance, thus, the values of the SUV cannot be a predictable factor for the presence of the genetic mutation. There was a statistically significant correlation in BRAF+ subgroup between the SUV values and the positive resection limit following surgery, showing a higher SUV value in the PET/CT evaluation. No correlation was observed between the aforementioned prognostic factors involved in papillary thyroid cancer and the BRAF V600E mutation.

## 1. Introduction

The BRAF mutation is associated with the oncogenesis of several tumors as well as colon cancer, melanoma and thyroid cancer [1]. In thyroid neoplastic pathology, the BRAF V600E mutation is shown to be involved in the oncogenesis of papillary thyroid cancer (PTC) and its subtypes [2]. Activation of the mutation in the mitogen-activated protein kinase (MAPK) pathway where the BRAF V600E gene is included, leads to the non-controlling tumor proliferation and thus, to the phenomenon of cell de-differentiation. Therefore, the presence of the mutation is associated with aggressive forms of the disease, the presence of an unfavorable prognostic factor and in some cases with the loss of avidity of tumor for the I-131 treatment [3,4]. Taking into account this scenario, the treatment option might be changed. One of these options is systemic therapy with tyrosine kinase inhibitors; numerous studies and clinical trials are ongoing on this topic, some of them with positive results and improvement of the long-term survival rate. In order to identify the cases with a high degree of aggressiveness and tumor de-differentiation, the correlation between gene mutation and the increased rate of carbohydrate metabolism assessed by positron emission tomography/computed tomography (PET/CT) hybrid imaging using F18-fluorodoxyglucose (F18-FDG) as a tracer, was studied [5,6,7,8,9]. 

The purpose of this study is to evaluate the correlation between the mutation of the BRAF V600E oncogene and the pathological standardized uptake values (SUV) at the PET/CT evaluation, for a group of patients with papillary thyroid cancer with persistent subclinical disease at 6 months after the initial radical treatment, consisting of surgery and radioiodine (I-131). We analyzed the correlations between the value of SUV and the presence of the BRAF mutation as well with other prognostic factors such as stage, age, extrathyroid extension, the presence of lymph nodes or distant metastasis, in order to identify those patients with aggressive outcomes and to personalize their therapies.

## 2. Patients and Method

### 2.1. Patients

This study includes 20 patients diagnosed with pure forms of papillary thyroid carcinoma, selected from a number of 663 new cases of thyroid carcinoma evaluated during 2019 in the Endocrine Tumor and Nuclear Medicine Department of the “Prof. Dr. Ion Chiricuţă” Institute of Oncology, a tertiary center, representing 3% of all treated patients. All patients were investigated, treated and evaluated uniformly in the same institution, a fact that suggests a unitary approach of this pathology.

All patients were evaluated after 6 months from the initial therapy during the monitoring program; the cases with persistent disease after the initial therapy were referred to F18-FDG PET/CT scans. The persistent disease was defined as follows: clinical, thyroid ultrasound and whole body I-131 scan all negatives, but with detectable serologic thyroglobulin (Tg) and/or pathologic levels of antibodies anti-thyroglobulin (anti-Tg) determined in stimulated conditions (elevated thyroid stimulating hormone-TSH).

The 20 selected patients with positive PET/CT scans were further investigated for BRAF V600E mutation. These patients had mean ± standard deviation (SD) age of 58.6 ± 13.35 years old, with a minimum 22 and maximum 74 years of age; there were 8 males and 12 females, with a ratio of 2:3. They had been radically treated with total thyroidectomy +/- lymphadenectomy, where this was necessary, and treated with one single administration of I-131; the mean ± SD I-131 activity was 82.2 ± 37.6 mCi (3.4 ± 1.39 GBq); all patients followed suppressive doses of Levothyroxine.

### 2.2. Genetic Analysis

The processing of samples for analysis of the BRAF V600E mutation was carried out within the Laboratory of Functional Genomics, Proteomics and Experimental Pathology of the same institution. For the isolation of genomic DNA, 5 sections of 10 μm thick paraffinated tissues were performed, which subsequently underwent the macrodissection technique. Genomic DNA was extracted using the Purelink Genomic DNA Mini kit (Invitrogen). For the quantification reaction of the BRAF V600E mutation through the real-time polymerase chain reaction (PCR) technique, it is essential to use a sufficient concentration of integrated DNA. As a result, the isolated DNA was quantitatively analyzed using the NanoDrop ® ND-1000 Thermo Scientific spectrophotometer, which determines the concentration and purity of the isolated genetic material. Thus, DNA can be measured directly in aqueous solutions by recording the absorption of that molecule at wavelengths of 230, 260, 280 nm, respectively, determining the difference between the amount of light transmitted to the sample and that received. In the case of pure DNA samples, the ratio A_260/_A_230_ must be within the 1.8–2.2 range, and in the case of the ratio A_260/A_/A_280_ the value of approximately 1.8 is accepted. The detection of the BRAF V600E mutation in the samples studied was undertaken by the real-time PCR technique using the IVD EntroGen BRAF Codon 600 Mutation Analysis kit II and the real-time PCR LightCycler 480 Roche. A concentration of DNA samples of 20 ng/μl was used for the PCR reaction. The evaluation of amplification products was carried out by reading two fluorochromes: FAM (483–533) for the BRAF V600E mutation and VIC (523–568) for internal control. 

### 2.3. Positron Emission Tomography/Computed Tomography (PET/CT) Imaging

Images were acquired with a GE Optima 560 PET/CT; F18-FDG PET/CT studies were performed after a period of fasting of 6 hours; the blood glucose levels, between 70 mg/dl and 150 mg/dl were measured before the F18-FDG injection; injected doses of 185–600 MBq F18-FDG were calculated accordingly to the patient’s weight and to the EANM procedure guidelines for tumor imaging, version 2.0 and the examination was performed after an uptake period of 60–90 minutes. CT images, with a slice thickness of 3.75 mm, were acquired using a low-dose protocol (100–120 Kv, 50–100 auto mA, index noise of 20%) in order to reduce the irradiation dose for patients. PET/CT images were evaluated by a team formed by a nuclear medicine physician and a radiologist. For all PET/CT studies, SUVlbm (the standardized uptake value lean body mass) was used as a semi-quantitative parameter for the F18-F18-FDG uptake calculation respecting a standard protocol on the work station (Volumetrix for PET/CT). All abnormal F18-FDG findings were correlated with neck ultrasound, serological tumor marker levels, and clinical examination of the patients. 

### 2.4. Serologic Analysis

Serum thyroglobulin (Tg) and thyroglobulin antibody (anti-Tg) measurements were carried out using immunochemical methods with electrochemoluminescence detection, Roche kits, and Cobas instruments (both from Roche Diagnostics, Basel, Switzerland), in the same accredited laboratory. Samples for Tg quantitation were taken after thyroid hormone withdrawal of ≥2 weeks with a TSH rise to 40 mIU/L. The Tg assay had a lower limit of detection of <0.04 µg/L, an intra-run coefficient of variation (CV) of 1.8%, and an inter-run CV of 3.0%. The TgAb assay had an intra-run CV of 5.6% and an inter-run CV of 8.7%. anti-Tg determinations <115 IU/mL were considered to be negative.

**Figure 1:**
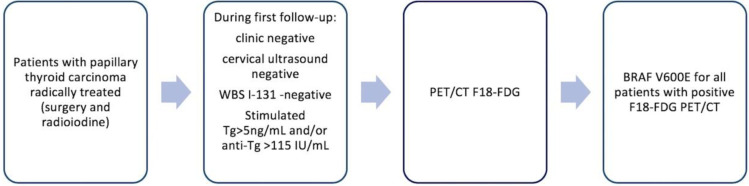
Diagnostic sequence for patients included in the study.

All patients signed the general institutional informed consent both for therapies and diagnostic procedures and for the possible use of anonymized data for scientific reports. The diagnosis protocol and decision criteria are highlighted in Figure 1.

### 2.5. Statistics

Data are presented as means ± standard deviations (SD), proportions, or both. Subgroup comparisons regarding characteristics and correlations were made using the Student’s *t*-test, Shapiro–Wilk test for uniform data and Spearman test for correlations. Statistical analyses were made using GraphPad Prism 6.0; P ≤ 0.05 was deemed to be significant.

## 3. Results

**Figure 2:**
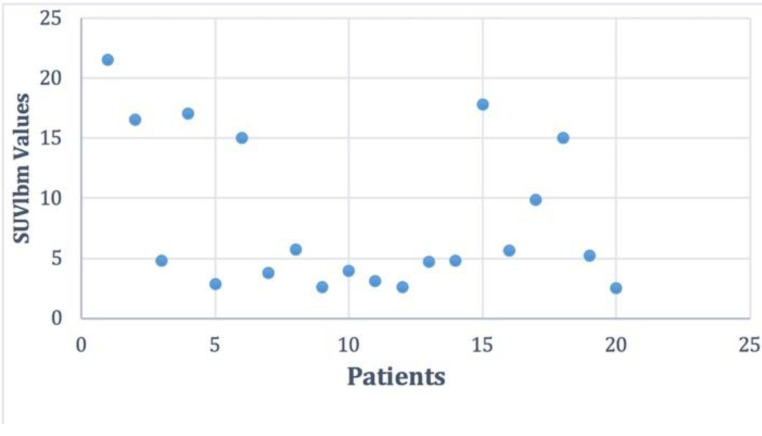
The distribution of the SUVlbm (standardized uptake value lean body mass) Max values in the studied cohort.

Considering previous published data [10] the number of patients with DTC and positive F18-FDG PET/CT scans represents 38%. For all patients with positive scans, we performed the BRAF mutation. Following the analysis of the BRAF V600E mutation, among the 20 patients, 10 of them had a positive result (50%), 9 a negative result (45%), and in one case the analysis was inconclusive. The distribution of SUVlbm Max values in the patient group was between Min and Max 2.5–21.57 with a mean ± SD of 8.25 ± 6.32 and is represented in Figure 2.

The mean ± SD SUVlbm value for BRAF+ (positive) patients was 8.99 ± 7.24 and it was higher than in the group BRAF– (negative) patients, where mean ± SD was 7.99 ± SD 5.64. 

The PET/CT evaluation reports detected pathological uptake of the tracer with suspicion of persistent disease as follows: latero-cervical lymph nodes in 8 cases (40%); thyroid bed in 3 cases (15%); both in the thyroid bed and at the cervical lymph node level in 2 cases (10%), and with suspicion of distant secondary metastases in lungs in 2 cases (10%), bones in 2 cases (10%), lung and bone in 1 case (5%) and mediastinum in 2 cases (10%). After distributing patients by stage and establishing a cut-off value of 55 years for the variable age, 75% of patients were over 55 years old; 7 patients (35%) were in advanced stages III and IVB. Prognostic factors, such as tumor stage, presence of metastatic lymph nodes or distant metastases, extrathyroid extension (EET), venous invasion (V), lymphatic invasion (L), and resection limit (R) were analyzed in Table 1. 

To analyze the statistical differences, between the BRAF+ and BRAF– subgroups and the prognostic factors, we performed the Student *t*-test; and the results are shown in Table 2.

In Table 2 we observe that none of the prognostic factors express a statistically significant difference between the two subgroups BRAF+ and BRAF–. The difference between SUV values is not statistically significant: ***P = 0.74 > 0.05;***

Regarding the specific tumor marker (Tg and anti-Tg) the Tg mean values ± SD in BRAF+ and BRAF– patients’ subgroups do not correlate statistically significantly: Tg 78.93 ± 142.6, respectively 74.03 ± 105.66; ***P = 0.93 > 0.05*.** With pathologic values of anti-Tg in the subgroup BRAF+ we had only 2 patients (4002 IU/mL and 181.7 IU/mL), and in the subgroup BRAF– only 1 patient with pathological values (anti-Tg – 917.4Iu/mL); also in this analysis ***P = 0.84 > 0.05*** and shows no statistical significance. 

Correlations of the BRAF variable were made with prognostic factors to verify the existence of correlations between them and the status of the genetic mutation (Table 3). After performing the correlation coefficients, we observed that the patient subgroup with BRAF V600E mutation did not have any statistically significant correlation with the variables studied, so the prognostic factors were not influenced by the status of the genetic mutation. 

Following the correlation between the stage and the rest of the prognostic factors, the results are presented in Table 4. 

A positive, statistically significant, medium-intensity correlation was identified between stage and age, so in the study group, patients with older age had a more advanced stage of the disease.

In the BRAF+ patients’ subgroup, we assessed the correlation of SUV values with the prognostic variables (Table 5).

Based on statistical analysis, in the BRAF+ subgroup we can say that the value of the SUV was statistically significant only by the resection limit. Thus, BRAF+ patients with a positive post-surgical resection limit had a higher SUV.

## 4. Discussion

It is well known that differentiated thyroid cancer, especially the papillary type, is a neoplastic pathology that generally has a very good prognosis, but there are also a few cases with less favorable evolution. The cases with serological persistent disease after the initial therapy (detectable Tg and antiTg) is a controversial issue. There is no consensus for the long-term clinical management of patients with DTC who have an elevated serum Tg or anti-Tg [11] and that is why the research in this field is open. In the present study we tried to focus on a cohort of patients with early serological persistent disease of classic papillary thyroid cancer, evaluated by F18-FDG PET/CT and BRAF mutation. According to published data, the subclinical persistent disease occurs in less than 15% and the positive scans for this group represents less than 40% [6,10]. From this group we selected the cases for mutation analysis. So, despite the large number of thyroid cancer patients, the low number of subjects taken in by the study was justified by the multiple criteria for selection and this is a limitation of our study. 

In the case of thyroid carcinomas arising from the follicle cells, the mutation of the BRAF and RAS genes are the most common; the BRAF gene mutation was considered present especially in papillary thyroid carcinomas [2]. The presence of this genetic mutation is associated with aggressive cases of thyroid carcinomas, with a low response rate to the iodine treatment and an unfavorable prognosis.

Analysis of the BRAF V600E mutation in patients with differentiated thyroid neoplasia is not undertaken routinely, because differentiated thyroid cancer (DTC), especially the papillary form, has a high rate of response to treatment. In some prospective studies published to date on the analysis of BRAF V600E mutation in patients with papillary thyroid carcinoma, the determination of the mutation was largely undertaken at the time of the total thyroidectomy surgical act or with the surgical reintervention for tumor relapse [12,13].

The PET/CT evaluation was carried out due to the suspicion of aggressive patterns of initial histology, tumor relapse based on elevated Tg values, as well as Anti-Tg values. Negative scanning after administration of I-131 correlated with positive values of tumor markers or their increase in dynamics, revealing an incomplete or even absent response to treatment and imposing a PET/CT evaluation. The contribution of the determination of BRAF V600E in these patients, and how the therapeutic behavior changes, is intensively debated and studied. Following genetic testing for the BRAF V600E mutation in the study group, in *n* = 10 patients (50%) the presence of the genetic mutation was identified; in 9 cases (45%) we found a negative result and in one patient the result was inconclusive due to insufficient DNA material. Among the selected patients with serological persistent disease after initial therapy at 6 months, only two patients had pathologic values of stimulated anti-Tg and had positive PET/CT scan and BRAF+; the statistical analysis showed that the values represent low-intensity negative correlation, which was statistically insignificant. Anti-Tg is considered a surrogate marker in PTC [13], thus the role in the definition of persistent disease should not be overlooked. 

We have correlated the BRAF mutation and prognostic factors such as age, sex, stage, presence of lymph node or distant metastases, presence of vascular or lymphatic invasion; no correlations between the presence of genetic mutation and the rest of the prognostic factors were revealed following statistical analysis. A similar result was also published for a population group in Korea, where the authors did not find any correlation between BRAF and the prognostic factors studied [14]. Also, a literature review and a multicenter study conducted on a group of patients in Italy concluded that the only association with the presence of the mutation was the advanced age of patients without the existence of any other correlation with a low prognosis in aggressive cases [9].

The two subgroups of BRAF+ and BRAF– patients were compared and no statistically significant differences were observed. More attention was paid to the SUV variable, since a significant positive correlation between the two variables is often associated. In our case, the value of SUVlbm Max for BRAF+ patients was higher, but without a statistically significant difference thus, the value of the SUV cannot be a predictable factor for the presence of the genetic mutation. In a work published in 2017, on a group of 107 patients the authors concluded that there was a correlation between the value of the SUV and the presence of the BRAF mutation, but in the more advanced tumor it was also correlated with the presence of its extrathyroid extensions and venous invasion [5,15]. The meta-analysis of Santhanam et al. [15] shows that the presence of the BRAF V600E mutation in PTC is related to a higher F18-FDG avidity and is associated with higher SUV uptake values compared to BRAF V600E mutation negative status. All 12 studies taken in the meta-analysis had a significant variation of different tumour types: follicular neoplasm, classic papillary thyroid cancer, poorly differentiated thyroid cancer, tall cell variant etc. The results of this meta-analysis compared with the present research which focused only on classic papillary forms, might be different due to this histological subtype of variation.

We also looked at how the SUV value was correlated with the rest of the prognostic variables. In the group of 20 patients, we identified a statistically significant correlation between the SUV value and the positive resection limit. In this way, patients with a positive resection limit following surgery had a higher SUV value following the PET/CT evaluation.

It is known that in the case of differentiated thyroid cancer the increased age is associated with a higher probability of death, this being influenced by the stage of the disease; therefore, in the present study, a positive correlation with statistical signification between the patient’s age and stage was revealed, which translates into the fact that patients taken into the study at a more advanced stage of the disease were older [16]. 

The number of cases with classic PTC and non-favorable outcome are very low; despite this clinical scenario the role of BRAF status is to select those who would benefit from a personalized approach as regards the treatment option, after PET/CT evaluation and determination of BRAF mutation. In our study the surgical intervention was addressed in 10 patients and systemic therapy with tyrosine kinase inhibitors (TKI) was addressed for 5 patients, 2 of them also having the BRAF mutation present. In fact, the evaluation of genetic mutations is applicable in the process of development of therapies targeting the production pathway [17,18,19]. In some studies, a better response rate to treatment has been observed in BARF+ patients, leading to the development of studies only for this category of patients [20,21,22,23,24]. So, even if we focus only on few patients, selecting their best personalized treatment option is important. Patients require careful monitoring during treatment, and not least monitoring of the response to treatment through serial PET/CT evaluations. 

## 5. Conclusions

The presence of BRAF V600E is associated with an increased risk of tumor relapse and a lack of response to iodine therapy. The value of SUV in the case of BRAF+ (positive) patients was higher than in the negative ones, but without statistical significance, thus, the value of the SUV cannot be a predictable factor for the presence of the genetic mutation. There was a statistically significant correlation between the SUV value and the positive resection limit following surgery, showing a higher SUV value in the PET/CT evaluation. No correlation was observed between the prognostic factors involved in papillary thyroid cancer and the BRAF V600E mutation. 

## Figures and Tables

**Table 1:** Characteristics of patients.

*No*	*Sex*	*BRAF* *status*	*SUV*	*Age* *yo*	*T*	*N*	*M*	*L*	*V*	*R*	*EET*	*Stage*
1	M	+	21.57	69	T4a	N1b	0	1	1	1	Yes	III
2	M	+	16.57	72	T3b	N1b	0	1	0	1	Yes	II
3	M	-	4.8	53	T3b	N1b	0	0	1	1	Yes	II
4	F	-	17.09	62	T2	N1b	0	1	0	0	Yes	II
5	F	+	2.86	58	T3b	N1b	0	0	0	0	Yes	II
6	F	+	15	60	T4a	N1b	0	1	1	1	Yes	III
7	F	-	3.76	39	T3a	N1b	0	0	0	0	Yes	I
8	M	+	5.7	41	T3b	N1b	0	1	0	1	Yes	I
9	M	+	2.6	63	T1a	N0	0	0	0	0	Yes	I
10.	F	-	3.94	61	T2	N1b	0	1	0	1	Yes	II
11.	F	N/a	3.11	62	T2	N1b	0	0	0	0	No	I
12.	F	+	2.56	66	T4a	N1b	0	0	0	1	Yes	III
13.	M	-	4.68	22	T3a	N1b	0	1	1	1	Yes	I
14.	F	-	4.82	71	T3b	N0	0	0	1	0	Yes	II
15.	F	-	17.78	72	T3b	N1b	1	1	1	1	Yes	IVB
16.	M	+	5.61	41	T2	N1a	0	1	1	0	No	I
17.	F	-	9.86	62	T3a	N1b	0	0	0	0	No	II
18.	F	+	15	59	T2	N0	1	0	0	1	No	IVB
19.	M	-	5.25	65	T3a	N0	1	1	1	0	Yes	IVB
20.	F	+	2.5	74	T1b	N0	1	0	0	0	Yes	IVB

SUV—standardized uptake value; T—tumor size; N—status lymph nodes; M—metastatic status; L—lymphatic invasion present or not; venous invasion present or not; R—resection margins; EET—extrathyroid extension; N/a—not available.

**Table 2:** Determination P in BRAF+ vs. BRAF–.

Parameter	SUV	Age	T	N	M	L	V	R	Stage	Tg	Anti-Tg	EET
Student *t*-test (P)	0.74	0.55	0.25	0.55	0.91	0.82	0.28	0.52	0.73	0.93	0.84	0.61

SUV—standardized uptake value; T—tumor size; N—status lymph node; M—metastatic status; L—lymphatic invasion present or not; V—venous invasion present or not; R—resection margins; EET—extrathyroid extension; Tg—thyroglobulin; anti-Tg—anti-thyroglobulin antibodies.

**Table 3:** Analysis of the correlation between **BRAF** status and the prognostic factors.

Variable	Correlation Coefficient	P	Interpretation
**SUV**	0.080	0.743	Low-intensity positive correlation, statistically insignificant
**Age**	0.148	0.543	Low-intensity positive correlation, statistically insignificant
**T**	0.270	0.262	Low-intensity positive correlation, statistically insignificant
**N**	–0.145	0.552	Low-intensity negative correlation, statistically insignificant
**M**	–0.027	0.911	Low-intensity negative correlation, statistically insignificant
**L**	–0.055	0.821	Low-intensity negative correlation, statistically insignificant
**V**	–0.258	0.285	Low-intensity negative correlation, statistically insignificant
**R**	0.155	0524	Low-intensity positive correlation, statistically insignificant
**EET**	–0.121	0.619	Low-intensity negative correlation, statistically insignificant
**Stage**	0.082	0.737	Low-intensity positive correlation, statistically insignificant
**Tg**	0.020	0.933	Low-intensity positive correlation, statistically insignificant
**Anti-Tg**	–0.049	0.839	Low-intensity negative correlation, statistically insignificant

SUV—standardized uptake value; T—tumor size; N—status lymph node; M—metastatic status; L—lymphatic invasion present or not; V—venous invasion present or not; R—resection margins; EET—extrathyroid extension; Tg—thyroglobulin; anti-Tg—anti-thyroglobulin antibodies.

**Table 4:** Analysis of the correlation between the **stage** of the disease and prognostic factors.

Variable	Correlation Coefficient	*p* Value	Interpretation
**SUV**	0.367	0.122	Low-intensity positive correlation, statistically insignificant
**BRAF V600E**	0.082	0.737	Low-intensity positive correlation, statistically insignificant
***Age***	*0.653*	*0.002*	***Positive medium-intensity, statistically significant correlation***
**T**	−0.147	0.547	Low-intensity negative correlation, statistically insignificant
**N**	−0.307	0.200	Low-intensity negative correlation, statistically insignificant
***M***	*0.806*	*0.001*	***Positive correlation of strong intensity, statistically significant***
**L**	−0.015	0.95	Low-intensity negative correlation, statistically insignificant
**V**	0.145	0.551	Low-intensity positive correlation, statistically insignificant
**A**	0.180	0.460	Low-intensity positive correlation, statistically insignificant
**EET**	−0.007	0.977	Low-intensity negative correlation, statistically insignificant
**Tg**	0.131	0.592	Low-intensity positive correlation, statistically insignificant
**Anti-Tg**	0.115	0.639	Low-intensity positive correlation, statistically insignificant

SUV—standardized uptake value; T—tumor size; N—status lymph node; M—metastatic status; L—lymphatic invasion present or not; V—venous invasion present or not; R—resection margins; EET—extrathyroid extension; Tg—thyroglobulin; anti-Tg—anti-thyroglobulin antibodies.

**Table 5:** Analysis of the correlation between the SUV and prognostic factors in the subgroup of patients with BRAF+.

Variable	Correlation Coefficient	*p*	Interpretation
Age	0.251	0.483	Low-intensity positive correlation, statistically insignificant
T	0.128	0.722	Low-intensity positive correlation, statistically insignificant
N	0.277	0.437	Low-intensity positive correlation, statistically insignificant
M	−0.017	0.96	Low-intensity negative correlation, statistically insignificant
L	0.566	0.087	Positive medium-intensity, statistically insignificant correlation
V	0.482	0.157	Low-intensity positive correlation, statistically insignificant
***R***	*0.666*	*0.035*	***Positive medium-intensity, statistically significant correlation***
EET	−0.095	0.793	Low-intensity negative correlation, statistically insignificant
Stage	0.316	0.372	Low-intensity positive correlation, statistically insignificant
Tg	0.025	0.943	Low-intensity positive correlation, statistically insignificant
Anti-Tg	−0.300	0.399	Low-intensity negative correlation, statistically insignificant

SUV—standardized uptake value; T—tumor size; N—status lymph node; M—metastatic status; L—lymphatic invasion present or not; V—venous invasion present or not; R—resection margins; EET—extrathyroid extension; Tg—thyroglobulin; anti-Tg—anti-thyroglobulin antibodies; *P*-statistically significant <0.05.
